# Dietary spray-dried plasma improves intestinal morphology of mated female mice under stress condition

**DOI:** 10.1186/s40781-018-0169-5

**Published:** 2018-06-04

**Authors:** Yanhong Liu, Jeehwan Choe, Sheena Kim, Byeonghyeon Kim, Joy M. Campbell, Javier Polo, Joe D. Crenshaw, James E. Pettigrew, Minho Song

**Affiliations:** 1 Department of Animal Science, University of California, Davis, CA USA; 2 Department of Animal Science and Biotechnology, Chungnam National University, Daejeon, South Korea; 3 APC Inc., Ankeny, IA USA; 4 Department of Animal Sciences, University of Illinois, Urbana, IL USA

**Keywords:** Intestinal morphology, Mated female mice, Spray-dried plasma, Stress

## Abstract

**Background:**

Stress causes inflammation that impairs intestinal barrier function. Dietary spray-dried plasma (SDP) has recognized anti-inflammatory effects and improvement of gut barrier function. Therefore, the purpose of this study was to investigate the effects of dietary SDP on intestinal morphology of mated female mice under stress condition.

**Results:**

Villus height, width, and area of small intestines were low on gestation day (GD) 3 or 4 under stress conditions, and higher later (Time, *P* < 0.05). Crypt depth of colon was low on GD 4 and higher later (Time, *P* < 0.05). Meanwhile, the SDP treatments improved (*P* < 0.05) intestinal morphology, indicated by increased villus height, villus width, villus area, and ratio between villus height and crypt depth of small intestines and crypt depth of colon, and by decreased crypt depth of small intestines, compared with the control diet. The SDP treatments also increased (*P* < 0.05) the number of goblet cells in intestines compared with the control diet. There were no differences between different levels of SDP.

**Conclusion:**

Dietary SDP improves intestinal morphology of mated female mice under stress condition.

## Background

Spray-dried plasma (SDP) is one of additives in swine diets [1–3] and contains high protein with balanced contents of amino acids and various physiological components, such as immunoglobulins, peptides, glycoproteins, and others [4], that contribute to modulation of microbial populations [5, 6], immune responses and inflammation [4, 7, 8], and intestinal morphology [8, 9], and others [4].

Based on these beneficial effects, SDP is typically used in nursery pig diets to maximize growth rate and to minimize health issues [1–3]. Previous research also showed that dietary SDP improved reproductive performance of sows under some stress conditions [10, 11] and pregnancy rate of mated female mice under transport stress [2].

Generally, various stressors can cause local or systemic inflammation [12–14], resulting in the impairment of intestinal barrier function [15–17] and detrimental effects on animal performance and health [18, 19]. It is well known that dietary SDP attenuates inflammation, strengthens intestinal barrier function, and improves intestinal morphology [9, 20–22]. Therefore, the objective of present study was to determine whether dietary SDP provides beneficial effects on gut health, mainly intestinal morphology, of mated female mice under transport stress.

## Methods

The experiment was conducted in the mouse facility of the Institute for Genomic Biology building at the University of Illinois at Urbana-Champaign, IL, USA. The mouse model of poor reproductive success used in this experiment was the same one used in the previous studies [2]. The levels of SDP (1 and 8%) in the diet were chosen for this experiment based on the results from the previous studies [2].

### Animals, housing, diets, and experimental design

A total of 156 mated female mice (C57BL/6 strain; 16.4 ± 1.1 g BW) were shipped from a vendor (The Jackson Laboratory, Bar Harbor, ME, USA) to the university facility (Urbana, IL, USA) on the day the vaginal plug was found (gestation day [GD] 1) and arrived at the IL facility on GD 3 after 2 d transport by air and ground. When the mice arrived at the facility, they were weighed and housed in individual cages with controlled temperature (23°C), humidity (40%), and a 12 h light and dark cycle. They were immediately and randomly assigned to dietary treatments (0, 1, and 8% SDP in the diet for CON, SDP1, and SDP8, respectively) and allowed free access to feed and water. The diets were formulated to meet or exceed NRC [23] estimates of nutrient requirements of mice and to have similar metabolizable energy, crude protein, amino acids levels, and no antibiotics (Table 1). The diets were pelleted without heating using a pellet press. The SDP was from bovine blood (APC, Inc., Ankeny, IA, USA).

**Table 1:** Ingredient composition of experimental diets

	Dietary treatments^a^		
Item	CON	SDP1	SDP8
Ingredients, %			
Dried skim milk	53.10	50.67	33.68
Corn starch	19.90	21.32	31.25
Sucrose	10.00	10.00	10.00
Spray-dried plasma^b^	–	1.00	8.00
Soybean oil	7.00	7.00	7.00
Cellulose	5.00	5.00	5.00
AIN-93 MX^c^	3.50	3.50	3.50
AIN-93 VX^d^	1.00	1.00	1.00
DL-methionine	0.25	0.26	0.32
Choline bitartrate	0.25	0.25	0.25
Calculated energy and nutrient levels			
ME, kcal/kg	3483	3492	3558
Crude protein, %	18.28	18.25	18.00
Ash, %	4.44	4.33	3.57
Ca, %	1.18	1.14	0.94
P, %	0.70	0.70	0.64

^a^CON = control diet; SDP1 = 1% spray-dried plasma diet; SDP8 = 8% spray-dried plasma diet

^b^The SDP was from bovine blood (AP 920; APC, Inc., Ankeny, IA, USA)

^c^Dyets, Inc., Bethlehem, PA, USA. Provided as milligrams per kilogram of diet: calcium, 5000; phosphorus, 1561; potassium, 3600; sodium, 1019; chloride, 1571; sulfur, 300; magnesium, 507; iron, 35; copper, 6; manganese, 10; zinc, 30; chromium, 1; iodine, 0.2; selenium, 0.15; fluorine, 1; cobalt, 0.5; molybdenum, 0.15; silicon, 5; nickel, 0.5; lithium, 0.1; vanadium, 0.1

^d^Dyets, Inc., Bethlehem, PA, USA. Provided per kilogram of diet: thiamin HCl, 6 mg; riboflavin, 6 mg; pyridoxine HCl, 7 mg; niacin, 30 mg; calcium pantothenate, 16 mg; folic acid, 2 mg; biotin, 0.2 mg; cyanocobalamin (vitamin B_12_), 25 μg; vitamin A palmitate, 4000 IU; vitamin E acetate, 75 IU; vitamin D_3_, 1000 IU; vitamin K_1_, 0.75 mg

### Sample collections and analyses

As an initial group, 12 randomly selected mice were weighed and euthanized by CO_2_ immediately after they arrived at our facility on GD 3. The rest of the mice were also weighed and euthanized by CO_2_; 12 mice per dietary treatment each day from GD 4 to 7. After euthanasia, the abdominal cavity was opened to collect intestine samples: duodenum, jejunum, ileum, and colon. For analysis of intestinal morphology, each section (3 cm) of intestine was fixed in 10% buffered formalin. The processing of intestine samples was based on the reports by Yi et al. [22] and Balan et al. [24]. The fixed samples were embedded in paraffin, sectioned at 5 μm-thickness, and stained with hematoxylin and eosin. The slides were scanned by NanoZoomer Digital Pathology System (Hamamatsu Co., Bridgewater, NJ, USA). All measurements of scanned slides were conducted using the associated NDP view software. Six straight and integrated villi and their associated crypts were selected to measure intestinal morphology (villus height, width, and area, crypt depth, ratio between villus height and crypt depth, and number of goblet cells).

### Statistical analyses

Data were analyzed using the PROC GLM procedure of SAS (SAS Inst. Inc., Carry, NC, USA) in a completely randomized design. The experimental unit was the mouse. For all measurements, the statistical model included effects of diet, time, and their interaction. In addition, pair-wise comparisons were performed among dietary treatments when a main effect of diet was found. The results are given as means ± SE. Statistical significance and tendency were considered at *P* < 0.05 and 0.05 ≤ *P* < 0.10, respectively.

## Results and discussion

**Fig. 1:**
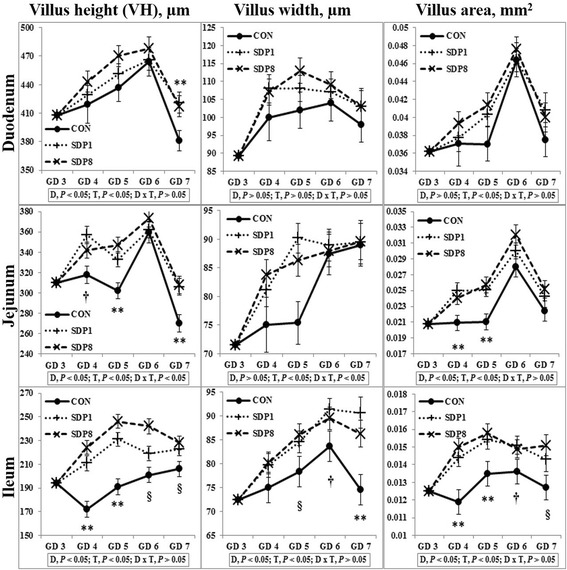
Villus height, width, and area in the small intestinal tract of mated female mice fed experimental diets. Values are means ± SE; *n* = 12 for GD 3, *n* = 7–12 for each treatment (GD 4 through GD 7); CON = control diet, SDP1 = 1% spray-dried plasma diet, SDP8 = 8% spray-dried plasma diet. D, diet; T, time; D x T, interaction between diet and time. **Different between CON and each other treatment (SDP1 and 8), *P* < 0.05. §Different between CON and SDP 8, *P* < 0.05. †Different between CON and SDP1, *P* < 0.05. No differences were found between SDP1 and SDP8

**Fig. 2:**
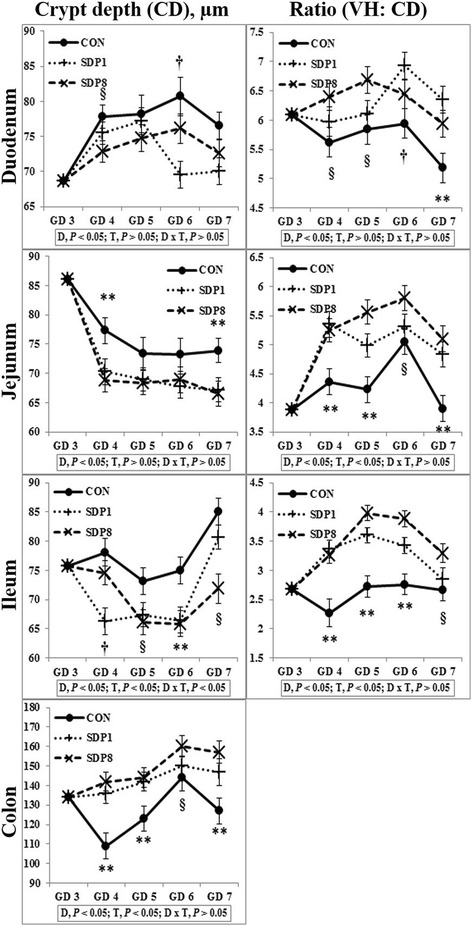
Crypt depth and ratio between villus height and crypt depth in the intestinal tract of mated female mice fed experimental diets. Values are means ± SE; n = 12 for GD 3, n = 7–12 for each treatment (GD 4 through GD 7); CON = control diet, SDP1 = 1% spray-dried plasma diet, SDP8 = 8% spray-dried plasma diet. D, diet; T, time; D x T, interaction between diet and time. **Different between CON and each other treatment (SDP1 and 8), *P* < 0.05. §Different between CON and SDP 8, *P* < 0.05. †Different between CON and SDP1, *P* < 0.05. No differences were found between SDP1 and SDP8

**Fig. 3:**
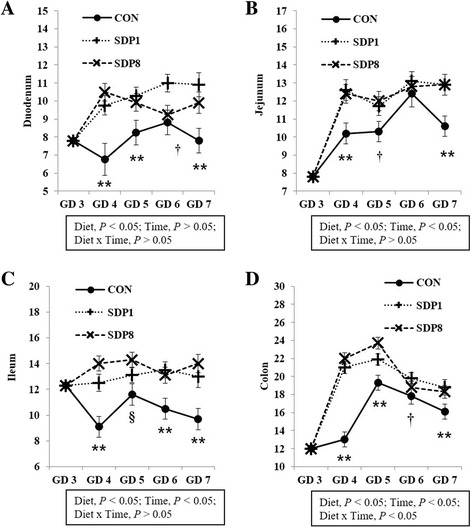
Number of goblet cells in the intestinal tract of mated female mice fed experimental diets. Values are means ± SE; n = 12 for GD 3, n = 7–12 for each treatment (GD 4 through GD 7); CON = control diet, SDP1 = 1% spray-dried plasma diet, SDP8 = 8% spray-dried plasma diet. (**a**) Duodenum, (**b**) Jejunum, (**c**) Ileum, (**d**) Colon. **Different between CON and each other treatment (SDP1 and 8), *P* < 0.05. §Different between CON and SDP 8, *P* < 0.05. †Different between CON and SDP1, *P* < 0.05. No differences were found between SDP1 and SDP8

Villus height, width, and area of all sections of small intestines were low on GD 3 or 4 under stress conditions, and higher later (Time, *P* < 0.05; except villus height of duodenum, Fig. 1). Crypt depth of colon was low on GD 4 under stress condition, and higher later (Time, *P* < 0.05), but there was no consistent time pattern of small intestinal crypt depth (Fig. 2). The SDP treatments improved (*P* < 0.05) intestinal morphology in all sections of intestines, indicated by increased villus height, villus width, villus area, and ratio between villus height and crypt depth of small intestines and crypt depth of colon and by decreased crypt depth of small intestines, compared with the CON (Figs. 1 and 2). The SDP treatments also increased (*P* < 0.05) the number of goblet cells in all sections of intestines compared with the CON (Fig. 3). There were no differences between SDP1 and SDP8 (Figs. 1, 2, and 3). Measurements of intestinal morphology showed that inflammation, perhaps caused by stress, diminished as over time after arrival. Stress may be detrimental to structural integrity of the intestine as shown by reduction of villus height and crypt depth [25–27], but may not influence it if stress is not acute [27–29]. Therefore, transport stress may have compromised the mucosal integrity at arrival. Meanwhile, feeding SDP improved intestinal morphology in the present experiment, in agreement with the results from previous studies using pigs under inflammation [20–22] and rats under normal conditions [24]. This benefit may derive from attenuation of inflammation by SDP, enhancement of gut barrier function by SDP, or both [9, 16]. The present experiment also shows that feeding SDP increases the number of goblet cells in all intestinal sections, in contrast to the lack of SDP effect in a previous study using pigs [30], suggesting a potential associated increase in mucin secretion and perhaps, therefore, in strengthening of the gut barrier function.

## Conclusion

Dietary SDP strengthened the integrity of intestinal morphology of mated female mice under stress condition.

## Abbreviations

GD: Gestation day
SDP: Spray-dried plasma.
